# Quinine for Hypodermatic Use

**Published:** 1898-09

**Authors:** 


Quinine for Hypodermatic Use. The best preparation of quinine for hypodermatic use is the hydrochlorate, containing as it does nearly 82 per cent. of alkaloidal quinine. This drug is soluble in 21 parts of water, and by the addition of 2 parts of antipyrine it becomes soluble in 2 parts of water. The following formula is recommended for hypodermatic use : ℞.—Quinine hydrochlorate (basic), 3 parts; antipyrine, 2 parts; distilled water, 6 parts. Mix and dissolve by the aid of a gentle heat. In order to prevent pain following the use of the above injection, a 2 or 3 per cent. solution of cocaine may be added.

